# Using buccal methylomic data to create explainable aging clocks as well as classifiers and regressors for lifestyle and demographic factors

**DOI:** 10.3389/fgene.2025.1637186

**Published:** 2025-10-01

**Authors:** Maxim N. Shokhirev, Adiv A. Johnson

**Affiliations:** Tally Health, New York, NY, United States

**Keywords:** smoking status, body mass index, alcohol intake, chronological age, aging clocks, DNA methylation, aging biomarkers, random forest

## Abstract

In human blood, it has been demonstrated that methylomic information can be used to predict smoking status, alcohol intake, and chronological age. While it is possible to robustly predict chronological age using DNA methylation information derived from buccal tissue, it remains to be determined if other variables can be directly predicted in cheek swabs. Here, we demonstrate that classifiers for smoking status and race/ethnicity can be built in a buccal methylomic dataset derived from 8,045 adults spanning an age range of 18–93 years. Furthermore, we build novel regressors for body mass index, alcohol intake, and chronological age. For each of these models, we identify the 1,000 most important CpGs and perform enrichment analyses on them to expose associated biological pathways and transcription factor targets. We additionally explore how the architecture of an epigenetic aging clock–specifically how many hidden layers are present–influences model accuracy. Finally, we build proof-of-concept, explainable deep learning models that connect DNA methylation sites annotated to genes to Reactome pathways or to transcription factors. These pathways and target sets are then used to estimate age, a feature that provides interpretability. All together, these findings further emphasize the usability of buccal data for epigenetic predictions.

## Introduction

It is well-established that methylomic data can be used to construct computational models that predict chronological age. These models–referred to as epigenetic aging clocks–can be broadly divided into first-generation clocks that are purely trained to predict chronological age and next-generation clocks that are trained in such a way that their output is associated with lifestyle, health, and/or age-related outcomes (Johnson and Shokhirev, 2025b). The buccal PedBE clock (McEwen et al., 2020) is an example of a first-generation, age-trained clock while bAge (Bernabeu et al., 2023), GrimAge2 (Lu et al., 2022), and CausAge (Ying et al., 2024) are all examples of more recent, next-generation clocks. Aging clocks have been widely used to investigate the relationship between myriad variables and epigenetic aging, unveiling clock associations with lifestyle factors, drugs, and supplements (Johnson et al., 2022).

While less studied, methylomic data can also be used to construct other types of predictors, including classifiers. In 2019, Bollepalli et al. built a machine learning classifier to detect smoking status in whole blood (Bollepalli et al., 2019). The feasibility of blood-based methylomic prediction of smoking status has also been demonstrated by Vidaki et al. (2023) and Fernandez-Carrion et al. (2023). Similarly, Liu et al. constructed a methylomic biomarker of alcohol consumption based on 144 CpG sites. In whole blood, this model was able to identify heavy alcohol intake with substantial accuracy (Liu et al., 2018). Very recently, the feasibility of an epigenetic predictor of smoking status in cheek swabs was also demonstrated (Pospiech et al., 2025).

Previously, we created a next-generation epigenetic aging clock called CheekAge that was optimized for prediction in adult buccal samples (Shokhirev et al., 2024). This model was trained to predict chronological age while also optimizing for a high correlation of the age residual with lifestyle/health factors using an ensemble of linear models. In addition to correlating with chronological age with a high accuracy, we showed that its delta age (epigenetic age–chronological age) was significantly associated with various demographic, lifestyle, and health factors after correcting for confounding factors such as cell type, sex, and chronological age. Indeed, the four most significant variables were race/ethnicity, body mass index (BMI), smoking status, and alcohol intake. Based on these prominent associations, we were curious if we could build novel, relevant predictors using buccal methylomic data. Here, we create novel classifiers for smoking and race/ethnicity as well as regressors for BMI, alcohol consumption, and chronological age. We additionally unveil top enrichment terms for the inputs of these models and evaluate how model architecture influences the accuracy of deep learning clocks. Finally, we build proof-of-concept explainable deep learning clocks that estimates age using Reactome pathways or transcription factor target gene sets, which are in turn connected to DNA methylation sites annotated to genes.

## Results

### Study overview

In this work, we leveraged our previously characterized buccal methylomic dataset derived from 8,045 adults spanning an age range of 18–93 years. This cohort was 51.9% male, 48.1% female, and ethnically/racially diverse. As described in more detail previously (Shokhirev et al., 2024), Infinium MethylationEPIC data was collected from each sample. In addition, this methylomic data was paired with answers from a questionnaire that provided lifestyle, health, and demographic information. The metadata along with all predicted values are included as Supplementary Table S1.

**FIGURE 1:**
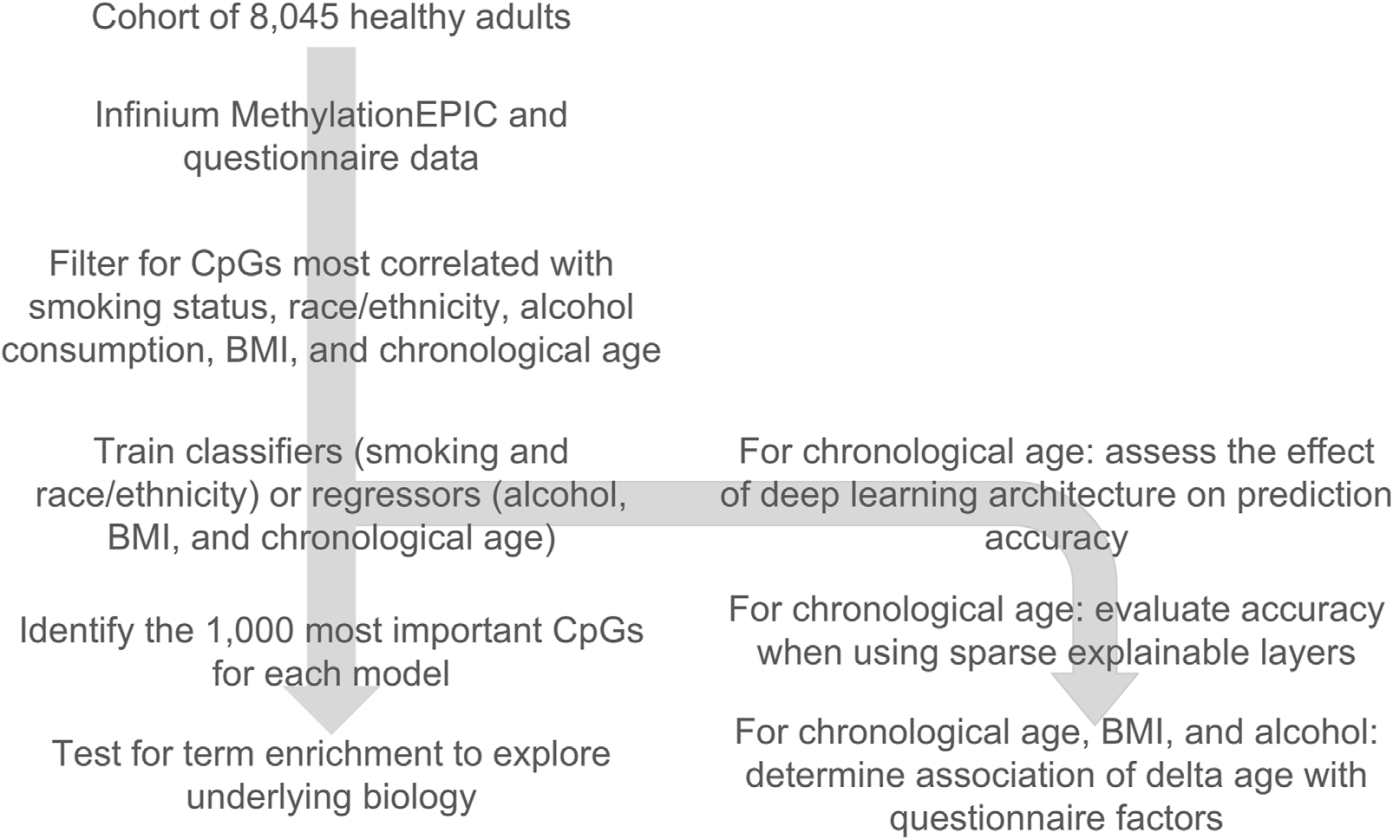
Overview of the design of the study. A cohort of 8,045 buccal Infinium MethylationEPIC methylomes coupled with a lifestyle, health, and demographic questionnaire was used to train classifiers and regressors of key lifestyle, health, and demographic variables. The top 1,000 important CpGs for each model were used to look for pathway and transcription factor binding target enrichment. For chronological age, the deep learning architecture of the model’s effect on accuracy was evaluated, sparse explainable deep learning models were developed, and the delta age (epigenetic age–chronological age) was used to check for lifestyle/health associations.

In our prior work, we demonstrated that the next-generation epigenetic aging clock CheekAge was most significantly associated with race/ethnicity, BMI, smoking status, and alcohol intake in this dataset. Motivated by these findings, we were curious if classifiers or regressors could be built for these variables. We additionally wanted to explore different approaches for the prediction of chronological age. In this work (Figure 1), we first filter for CpGs that are the most correlated with race/ethnicity, BMI, smoking status, alcohol intake, and chronological age, demonstrate that no significant batch effects are present (Supplementary Table S2), and then train classifiers for smoking and race/ethnicity as well as regressors for alcohol, BMI, and chronological age. For chronological age, we additionally assess the effects of deep learning architecture on prediction accuracy and evaluate accuracy while using sparse explainable layers. We further assess the ability of a chronological age-trained deep learning model to associate with lifestyle and health variables. Finally, we identify the 1,000 most important CpGs for these models and explore enrichment results for Reactome pathways and transcription factor gene targets.

### Classifiers for smoking status and race/ethnicity

**FIGURE 2:**
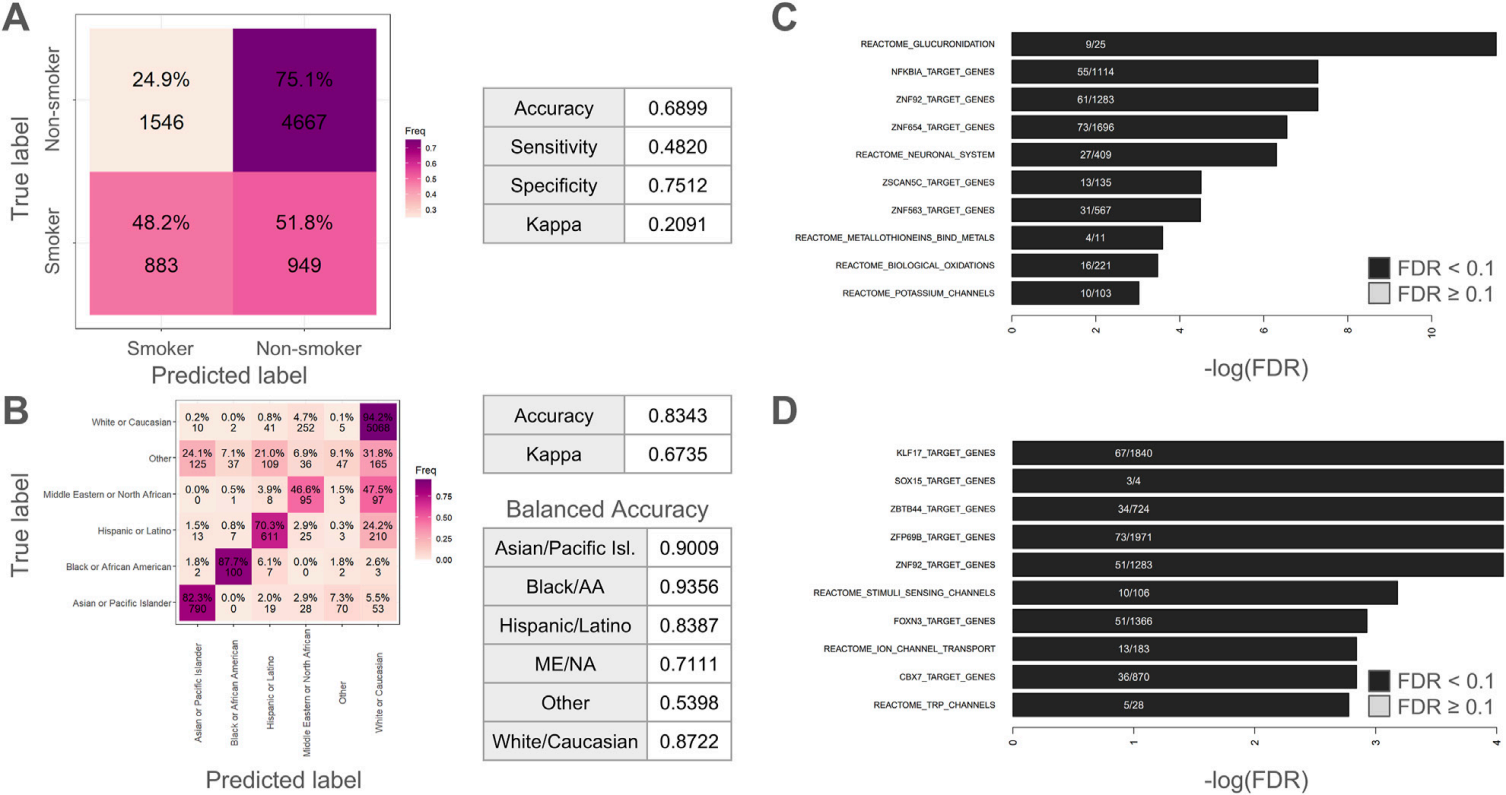
Classifying smoking status and race/ethnicity using random forest classifiers. **(A)** Confusion matrix for smoking status classifier. **(B)** Confusion matrix for race/ethnicity classifier. Tables show relevant accuracy metrics. **(C–D)** Enrichment analysis on the 1,000 most important methylation sites showing top 10 hits for **(C)** smoking or **(D)** race/ethnicity. ME/NA = Middle Eastern or North African. Black/AA = Black or African American.

We began by training random forest classifiers for smoking status (Figure 2A) and race/ethnicity (Figure 2B). The classifier for smoking status was mediocre with an overall accuracy of 0.6899, a sensitivity of 0.4820, a specificity of 0.7512, and a Kappa of 0.2091. These metrics suggest that the classifier is much more adept at determining when someone is not a smoker versus is a current smoker. The kappa value is indicative of fair agreement between predicted and actual classifications and conveys that the classifier is doing better than random chance (which would be indicated by a Kappa value of zero). Surprisingly, the classifier for race/ethnicity (Figure 2B) was considerably more accurate with an overall accuracy of 0.8343 and a Kappa of 0.6735 (indicating substantial agreement between predicted and actual classifications). In terms of specific ethnic/racial groups, the balanced accuracy was 0.9009 for Asian or Pacific Islander, 0.9356 for Black or African American, 0.8387 for Hispanic or Latino, 0.7111 for Middle Eastern or North African, 0.8722 for White or Caucasian, and 0.5398 for Other Racial/Ethnic groups.

For the enrichment analysis of top smoking status CpGs, the top 1 0 Reactome and transcription factor target results are visualized in Figure 2C. Among these top 10 results, the Reactome pathways “Glucuronidation”, “Neuronal System”, “Metallothioneins bind metals”, “Biological oxidations”, “Potassium Channels”, were all significantly enriched. The top result was “Glucuronidation”, which refers to a critical drug metabolism and clearance pathway in the human body (Miners and Mackenzie, 1991). As described in the Reactome database (Milacic et al., 2024), the Reactome pathway “Biological oxidations” is pertinent to the processing of foreign chemicals. The metal-oriented result “Metallothioneins bind metals” is interesting given that various heavy metals can be found in tobacco smoke (Caruso et al., 2013). Likewise, the second-most enriched term was “NFKBIA Target Genes”. This is intriguing given that cigarette smoke has been reported to induce NF-kappaB activation in human immune cells (Hasnis et al., 2007). As such, these results make intuitive sense for a smoking classifier. The results for the race/ethnicity classifier (Figure 2D) were more difficult to interpret and included the significantly enriched Reactome pathways “Stimuli sensing channels” and “Ion channel transport” as well as enrichment for transcription binding targets for *KLF17*, *SOX15*, *FOXN3*, *CBX7*, and various zinc finger transcription factors. The top enriched terms are tabulated in Supplementary Table S3.

### Regressors for alcohol intake, BMI, and chronological age

**FIGURE 3:**
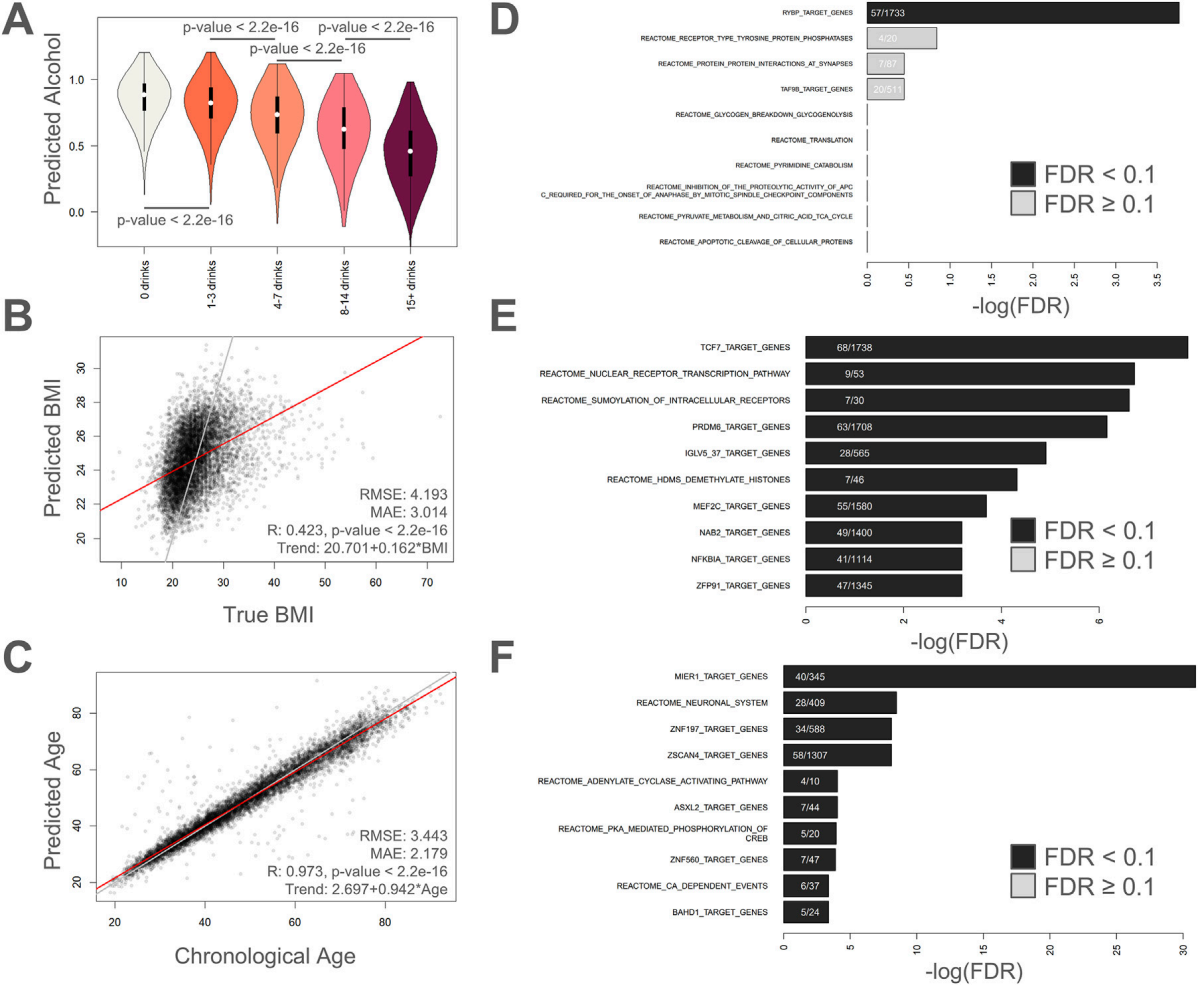
Regressing on alcohol intake, BMI, and chronological age. **(A)** Violin plots showing distribution of predicted alcohol abstinence (1 = no alcohol, 0 = 15+ drinks) for different categories of answers about alcohol consumption. Bars with p-values indicate significant differences using Welch’s T-test. Violin plot colors are different to help differentiate the different categories only. **(B–C)** Scatter plots showing predicted values compared to actual values for **(B)** BMI, and **(C)** chronological age. RMSE: Root mean squared error, MAE: mean absolute error, R: Pearson’s correlation. **(D–F)** Enrichment analysis on the 1,000 most important methylation sites showing the top hits for **(D)** alcohol intake, **(E)** BMI, and **(F)** chronological age.

In Figure 3, plots visualizing predicted values versus actual values are shown for alcohol intake (Figure 3A), BMI (Figure 3B), and chronological age (Figure 3C). For the alcohol intake regressor (Figure 3A), we see a gradual but highly significant increase in predicted alcohol intake (lower predicted alcohol score) with increasing self-reported weekly alcohol consumption. The Pearson correlation, MAE, and RMSE values for the BMI regressor were 0.423 (p-value <2.2e-16), 3.014, and 4.193, respectively (Figure 3B). For the chronological age regressor (a new first-generation epigenetic aging clock based on the top 10,000 age-correlated CpGs and trained using deep learning), the Pearson correlation was 0.973 (p-value <2.2e-16), the MAE was 2.179, and the RMSE was 3.443.

The only significant enrichment result for the alcohol intake regressor was “RYBP target genes” (Figure 3D). The gene RYBP encodes for RING1 and YY1-binding protein (UniProt, 2025) and has been implicated in stem cell differentiation (Ujhelly et al., 2015), tumor suppression (Tan et al., 2017), and homologous recombination repair (Ali et al., 2018). For BMI, the following three Reactome pathways were significantly enriched: “Nuclear Receptor transcription pathway”, “SUMOylation of intracellular receptors”, and “HDMs demethylate histones” (Figure 3E). The first two results implicate cellular signaling while the latter results highlight epigenetic regulation and gene activity. The Reactome pathways “Neuronal System”, “Adenylate cyclase activating pathway”, “PKA-mediated phosphorylation of CREB”, and “Ca-dependent events” were all significantly enriched by inputs of the epigenetic aging clock (Figure 3F). The results highlight a clear theme of cellular signaling. In addition, the top hit was “MIER1 target genes”, which is a potential histone chaperone (Wang et al., 2023) that was recently implicated in liver regeneration (Xiong et al., 2024). The “ZSCAN4 target genes” is also intriguing given the role that ZSCAN4 plays in telomere maintenance (Meltzer et al., 2024). The top enriched terms are tabulated in Supplementary Table S3. We also checked for gene enrichment, CpG island architecture enrichment, and genomic regulatory group enrichment for the top 1,000 important age CpGs (Supplementary Table S4). The top 10 enriched genes for the top 1,000 age CpGs were *ZNF154*, *KLF14*, *FOXG1*, *EPHA7*, *ATXN8OS*, *STXBP5L*, *NEFM*, *KLHL1*, *ZNF701*, and *ZIC1*. Furthermore we saw considerable enrichment for CpG islands (FDR = 4.48E-268), and “Unclassified_Cell_type_specific” (FDR = 3.20E-33) and “Unclassified” (FDR = 1.42E-26) regulatory groups (Supplementary Table S4).

While we show values predicted on held-out samples, it is important to demonstrate that the predictors also work in separately collected datasets. To this end, we used a previously published 225 sample buccal methylation dataset spanning an age range of 18–100 years. Importantly, this dataset contained alcohol consumption, age, race/ethnicity, BMI, and smoking metadata (Shokhirev et al., 2024). We found that the age regressor has excellent performance with an RMSE of 3.84 years and an R of 0.972 (Supplementary Figure S1A). The alcohol predictor showed significant differences for the no alcohol group compared to those that have had five or more drinks in a single day in the past year, 4-7 drinks per week, and 15+ drinks per week, with a weaker difference compared to those that drink 8–14 drinks each week (Supplementary Figure S1B). The BMI regressor had similar performance in this validation dataset as well, with an R of 0.380 (p-value = 3.84e-9) and an RMSE of 4.226 (Supplementary Figure S1C). The smoking classifier showed similar accuracy metrics in this validation dataset compared to the original dataset with an overall accuracy of 0.7111 and a Kappa of 0.2091 (Supplementary Figure S1D). Finally, the race/ethnicity classifier, while still accurate, was slightly less robust in this validation dataset with an overall accuracy of 0.7511 and a Kappa of 0.5254 (Supplementary Figure S1E). All of the metadata for this dataset is included in Supplementary Table S5.

**FIGURE 4:**
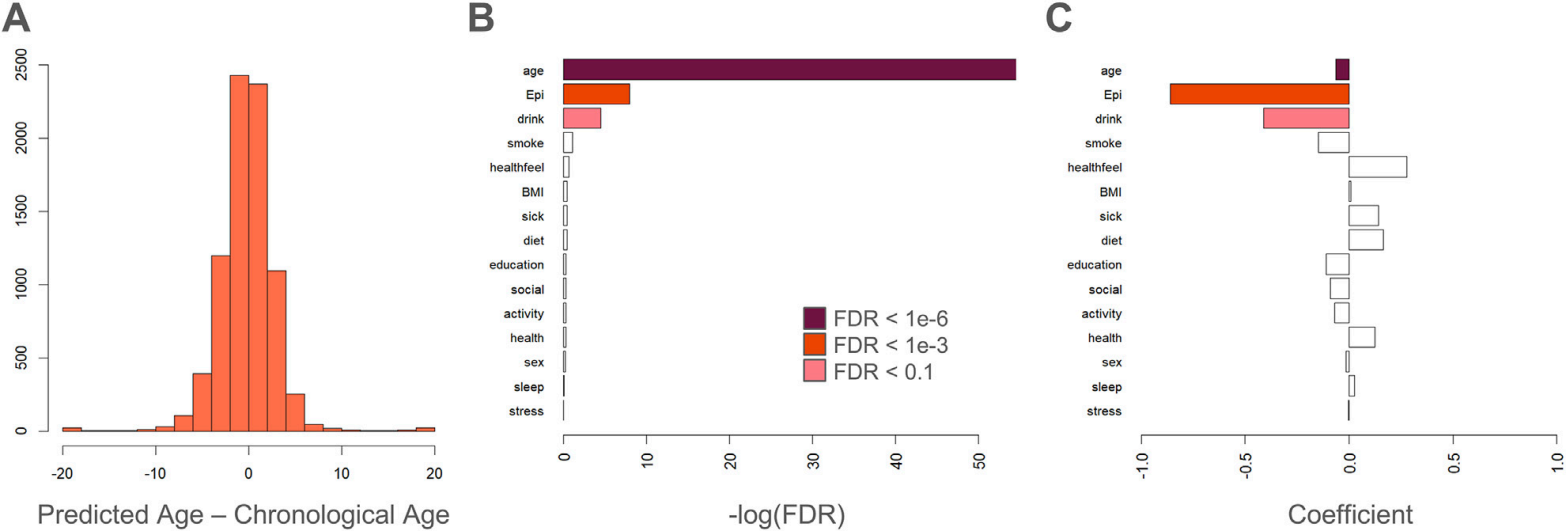
Age regressor delta poorly associates with lifestyle or health. **(A)** Histogram showing delta ages: epigenetic ages (predicted by the deep learning model in Figure 3C) - chronological ages. **(B)** Bar plot showing the negative log of the FDR for each fit coefficient in a linear model of delta age. Delta age is associated significantly with chronological age, estimated epithelial fraction, and alcohol consumption. **(C)** Directionality of each association is indicated by best fit coefficients for each variable in a linear model of delta age.

Previously, we showed that the next-generation epigenetic aging clock CheekAge (a model that was trained using simulated annealing and uses clustering and ensembling in conjunction with more than 200,000 CpG inputs) is associated with the majority of lifestyle and healthy factors in this dataset. Here, we explore the ability of this newly created epigenetic aging clock (Figure 3C) to associate with these variables. Although an expected spread of delta ages (epigenetic ages–chronological ages) was seen for this model (Figure 4A), only chronological age, estimated epithelial fraction, and alcohol intake were significantly associated (Figure 4B). The significance of the alcohol association was relatively weak (FDR <0.1) and directionality for each association is shown in Figure 4C. These findings provide further evidence that optimizing for chronological age predictions alone is insufficient for a clock’s ability to associate with meaningful health and lifestyle variables. Indeed, we previously showed that simple, first-generation clocks trained using strategic inputs that are linked to health and disease signals similarly fail to broadly associate with lifestyle and health signals (Johnson and Shokhirev, 2025a).

In addition to delta age associations, we checked for linear associations between predicted smoking, predicted BMI, and predicted alcohol consumption with other variables. Unsurprisingly, we found that predicted smoking, predicted BMI, and predicted alcohol consumption are each significantly linearly associated with the reported smoking, BMI, and alcohol consumption (Supplementary Figures S2A-C). We also found that these predicted values are associated with other variables such as chronological age, sex, education levels, epithelial cell fraction, and others, although the magnitude of the effect was typically relatively low (Supplementary Figures S2D-F). Linear model coefficients and significance values are included in Supplementary Table S6.

### The impact of model architecture on chronological age prediction

Given the impressive correlation exhibited by the model in Figure 3C, we were curious how models with distinct architecture would compare. To assess this, we built a simplified model with direct linear connections to chronological age (Supplementary Figure S3A), a model with a single 2,000 node hidden layer (Supplementary Figure S3B), a model with a 2,000 node hidden layer and an additional 500 node hidden layer (Supplementary Figure S3C), and a model with three hidden layers–one 2,000 node, one 500 node, and one 50 node (Supplementary Figure S3D). The least accurate model was the one that did not contain a hidden layer and displayed a Pearson correlation of 0.796, a MAE of 7.160, and an RMSE of 8.996 (Supplementary Figure S3A). Accuracy notably increased with the inclusion of a single hidden layer (Supplementary Figure S3B) and appeared to peak when two or more layers were included. Specifically, the two-layer model (Supplementary Figure S3C) showed a Pearson correlation of 0.973, a MAE of 2.179, and an RMSE of 3.443. The three-layer model (Supplementary Figure S3D) was comparable but slightly less accurate with a Pearson correlation of 0.971, a MAE of 2.251, and an RMSE of 3.515. While all four models were trained using the same 10,000 most correlated age CpGs, the top 1,000 most important CpGs for each model revealed that there was significant overlap in the top 1,000 most important CpGs, but the three models with hidden layers shared more common top important CpGs (Supplementary Figure S4A; Supplementary Table S7). In addition to demonstrating that various models can be built to predict chronological age with high accuracy in buccal methylomic data, these data demonstrate that accuracy and how CpG inputs are used is sensitive to model architecture, particularly the number of hidden layers included in the model.

### An explainable deep learning model

Recently, there has been a push to develop epigenetic aging clocks that are interpretable (Teschendorff and Horvath, 2025), meaning that some level of explanation is provided as to why a given epigenetic age was produced. Indeed, recent efforts have shown that it is possible to build epigenetic aging clocks that contain some degree of inherent explainability. For example, models can be built on epigenetic proxies of explainable proteins (Lu et al., 2022) or exploit explainable biological pathways connected to DNA methylation sites annotated to genes (Prosz et al., 2024).

**FIGURE 5:**
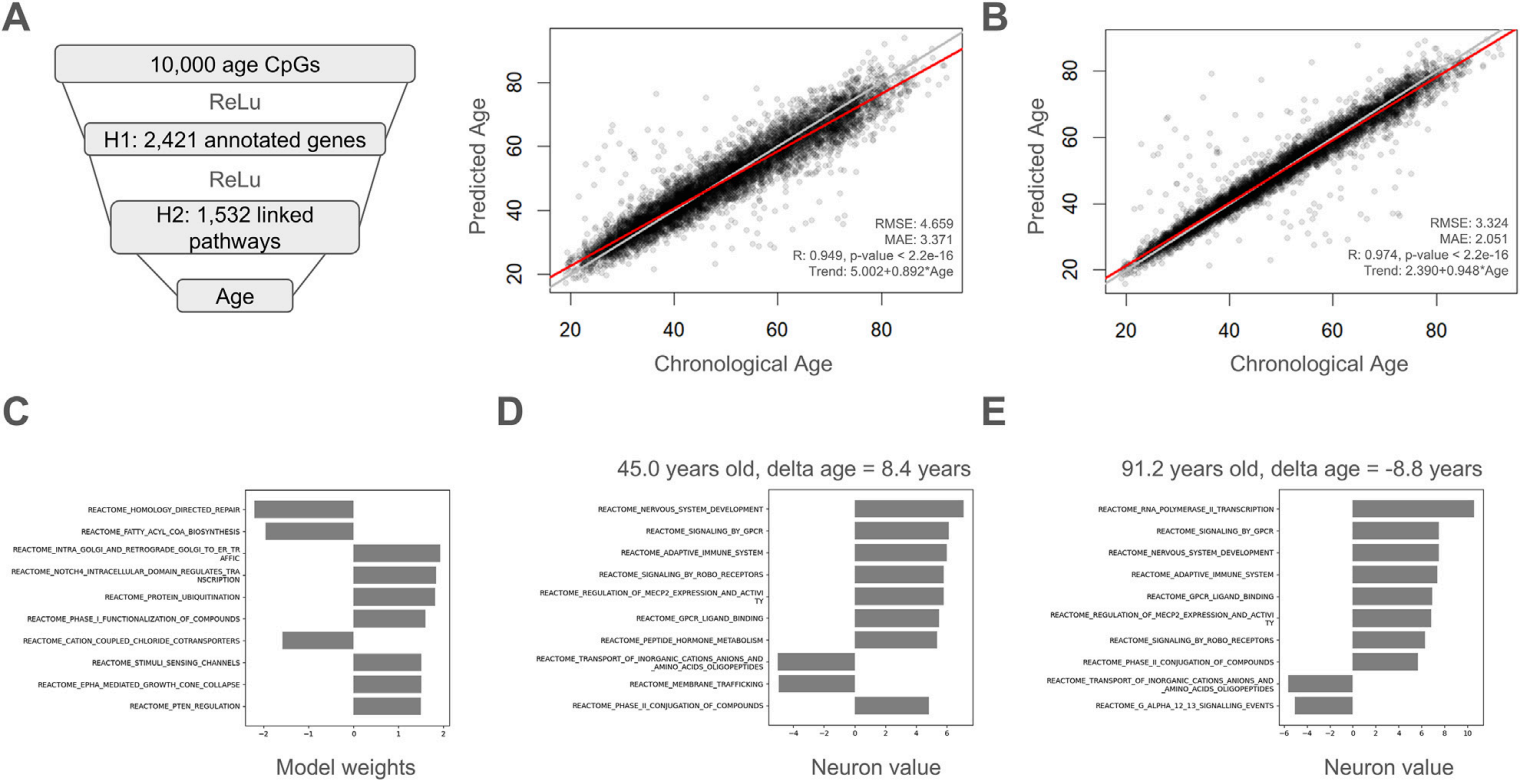
A proof-of-concept explainable deep learning model. **(A)** Chronological age-correlated CpGs are connected to a hidden layer of genes annotated to those CpGs. Genes are connected to the Reactome pathways they are a part of. Pathways are then used to estimate chronological age. The model was trained using a five-fold cross validation approach and held-out test values are plotted and evaluated for accuracy compared to chronological age. **(B)** A fully connected, unexplainable version of the model in **(A)** was trained for comparison using a five-fold cross validation approach and held-out test values are plotted and evaluated for accuracy compared to chronological age. RMSE: Root mean squared error, MAE: mean absolute error, R: Pearson’s correlation. **(C)** Top weighted pathways in the final explainable model. **(D)** Example showing calculated weights for top pathways for a sample with a high delta age of +8.4 years and a chronological age of 45.0 years. **(E)** Example showing calculated weights for top pathways for a sample with a low delta age of −8.8 years and a chronological age 91.2 years.

Here, we used age-related CpGs that were connected to a hidden layer of genes annotated to those DNA methylation sites. These genes were connected to Reactome pathways and these pathways were then used to estimate chronological age. Of the 10,000 age-correlated CpGs, 2,421 had gene annotations and there were 1,532 pathways linked to these annotated genes (Figure 5A). The resulting proof-of-concept explainable model was quite accurate with a Pearson correlation of 0.949, a MAE of 3.371, and an RMSE of 4.659. For comparison, we trained a non-explainable version of this model with the same number of fully connected neurons in each layer and noticed that imposing a biology-guided sparse model architecture does result in a modest drop in accuracy (Figures 5A,B). The top weighted pathways for this explainable model are highlighted in Figure 5C and were ʺHomology Directed Repairʺ, ʺFatty acyl-CoA biosynthesisʺ, ʺIntra-Golgi and retrograde Golgi-to-ER trafficʺ, ʺNOTCH4 Intracellular Domain Regulates Transcriptionʺ, ʺProtein ubiquitinationʺ, ʺPhase I - Functionalization of compoundsʺ, ʺCation-coupled Chloride cotransportersʺ, ʺStimuli-sensing channelsʺ, ʺEPHA-mediated growth cone collapseʺ, and ʺPTEN Regulationʺ. Examples of explainable results are shown for a sample with a high delta age of +8.4 years (Figure 5D) and a separate sample with a low delta age of −8.8 years (Figure 5E). Interestingly, the pathway “Adaptive Immune System” had a higher neuron value in the individual with a negative delta age (Figure 5E) than the individual with a positive delta age (Figure 5D).

Next, we tested if a similar explainable model can be trained using the transcription factor target gene sets instead of the Reactome pathway gene sets. Importantly, while this explainable model included a smaller number of gene sets (1,137 compared to 1,532 Reactome gene sets), the accuracy was somewhat better (Supplementary Figure S5A), though not as robust as the fully connected, non-explainable version of this model (Supplementary Figure S5B). Dissecting the model weights, we see that *UBN1*, *ZNF513*, *LEF1*, *SRF*, *PIAS4*, *MEF2*, *NRF2*, *HAND1*, *ATF6*, and an unknown GGGYGTGNY motif comprise the top weights (Supplementary Figure S5C). The ATF6 transcription factor is well known for its role in mediating the unfolded protein response and ability to influence organismal lifespan (Burkewitz et al., 2020). Looking at the same two samples with a delta age of +8.4 years (Supplementary Figure S5D), and −8.8 years (Supplementary Figure S5E), we notice that while many of the top transcription factor target neurons are the same, there are marked differences. For example, *ZNF320* is one of the top 10 results for the −8.8 delta age sample, while *AREB6* is in the top 10 for the +8.4 delta age sample. Although very little is known about *ZNF320*, this gene encodes for Zinc finger protein 320 and has been linked to both cell cycle and immunity. Moreover, the methylation status of *ZNF320* has been shown to correlate with overall survival in patients with hepatocellular carcinoma (Zhen et al., 2022). *AREB6*, which is also known as *ZEB1*, encodes for Zinc finger E-box-binding homeobox 1 and behaves as a transcriptional repressor. The methylation status of this transcription factor has also been linked to prognosis in patients with colon cancer (Fernandez-De-Los-Reyes et al., 2023).

In addition, when looking at the top 1,000 important CpGs for the explainable models and the fully-connected non explainable control models (Supplementary Figure S4B), we noticed that the explainable models included mostly unique top CpGs (618 for Reactome and 592 for transcription factor targets), while sharing 121 unique CpGs between themselves, and then 54 and 50 CpGs with AgeLinear1, the deep learning model with no hidden layers, and the other models, to a smaller degree. Of note, when looking at the top 1,000 important CpGs for CheekAge (Shokhirev et al., 2024), only 159 of the CpGs were shared with any of the other models we developed in this work (Supplementary Table S7). Nevertheless, given that CheekAge was trained on 211,003 CpGs and only 2,738 of those CpGs overlapped the top 10,000 CpGs correlated with age, there is a significant CpG overlap between top CheekAge CpGs and any of the other age models (p-values <2.143 e-1 1, hypergeometric test).

## Concluding remarks

In this work, we show that buccal methylomic data can be used to build novel classifiers for smoking status and race/ethnicity as well as new regressors for weekly alcohol intake, BMI, and chronological age. In addition to demonstrating feasibility, this creates the possibility of imputing missing data in other buccal datasets lacking this information. All classifiers and regressors and associated code can be accessed in Mendeley Data (DOI: 10.17632/m4zjkxss8f.2). Future efforts are warranted to determine if accuracy could be improved further to help increase the value of the imputed data. Moreover, the approach utilized here showcases how novel biological insights can be gained based on methylomic classifiers and regressors.

In addition, we saw that a first-generation model trained to predict chronological age alone did not linearly associate with most lifestyle and health variables, reminiscent of our earlier findings using this dataset (Shokhirev et al., 2024; Johnson and Shokhirev, 2025a). It is becoming increasingly clear that, in order to create an epigenetic clock that is highly associated with variables relevant to aging, a strategic, multi-step training process is needed. Indeed, notable differences in performance have been observed between first-generation clocks and more sophisticated, next-generation models (Levine, 2020; Crimmins et al., 2024). It is also worth noting that confounding variables such as predicted epithelial cell type proportion may play a small but significant role in the predicted age difference, and should be included when testing for linear association significance when possible.

Likewise, we demonstrate that it is possible to create an explainable deep learning model in buccal methylomic data. Specifically, we show that Reactome pathways or transcription factor gene target sets can be used as explainable inputs based on DNA methylation sites with gene annotations. Theoretically, a model’s interpretability could be further enhanced by only picking pathways that are easier to understand and/or tangibly connected to aging hallmarks (Lopez-Otin et al., 2023). Using the example of the older individual with a chronological age of 91.2 years and an epigenetic age of 82.4 years, the “Phase II - Conjugation of compounds” result with a positive neuron value is clearly relevant to detoxification. In contrast, the “G alpha (12/13) signalling events” result with a negative neuron count is more challenging to understand. In the Reactome database, there are higher-order, more understandable pathways such as “Innate Immune System”, “Metabolism”, and “Cellular Senescence”. It would be worthwhile to determine if a deep learning model could be built based on these higher-order, more comprehensible pathways. Ideally, such a model would also be trained in a way to maximize its ability to associate with lifestyle, health, and age-related outcomes. The result of these efforts would be an explainable, next-generation epigenetic aging clock.

To date, the overwhelming majority of epigenetic aging clocks published have been black boxes that lack interpretability. The most common way for an epigenetic clock to be trained is to use elastic net regression to identify a set of CpG inputs that can be linearly combined to predict epigenetic age. This is, for example, how the first-generation Hannum 2013 (Hannum et al., 2013), Horvath 2013 (Horvath, 2013), Horvath 2018 (Horvath et al., 2018), and PedBE (McEwen et al., 2020) models were created. Starting in 2018, several different next-generation models have been generated using multi-step training approaches. Some of these, like DNAm PhenoAge (Levine et al., 2018) and DunedinPACE (Belsky et al., 2022), fully lack explainability. Other next-generation models do include pseudo-explainability by first generating epigenetic proxies to predict other biomarkers. For example, GrimAge (Lu et al., 2019), GrimAge2 (Lu et al., 2022), and DNAm FitAge (McGreevy et al., 2023) all use epigenetic proxies (of either circulating proteins or fitness metrics) as model inputs. While much is still unknown, a given output can be better understood by this layer of proxies. Other recent models, such as PathwayAge (Li et al., 2025) and XAI-Age (Prosz et al., 2024), are similar to the Reactome-based explainable deep learning model we present here in that they also use pathways to offer explainability. While one of our models and XAI-AGE both focus on Reactome, PathwayAge abstracts CpGs into KEGG (Kanehisa et al., 2023) or Gene Ontology (Gene Ontology et al., 2023) pathways. All of these newer, pathway-level explainable models are quite adept at predicting chronological age and exhibit comparable accuracy. It remains to be determined just how adept these newer models are at capturing biological insights, but recent work involving PathwayAge (Li et al., 2025) suggests that a clock can be developed that is explainable, accurate, and significantly associated with age-related disease.

The field of epigenomics is evolving at a rapid pace and it is becoming increasingly clear that methylomic data can be used to predict a multitude of factors. As technologies provide access to more and more of the methylome and as methylomic datasets continue to grow, it remains unknown just how accurate these predictors can be. Furthermore, the field is likely just beginning to scratch the surface of predictive power in terms of algorithmic approaches that are both functionally relevant and explainable.

## Methods

### Cohort selection and survey

We used a previously published buccal dataset which consisted of 8,045 samples collected from healthy diverse adults residing in the United States of America (Shokhirev et al., 2024). For each of the 8,045 samples, we collected responses to 11 lifestyle- and health-related questions. Smoking status was determined based on whether a user had smoked over 100 cigarettes or was actively smoking (n = 1,832). Race/ethnicity was self reported as: Asian or Pacific Islander (n = 960), Black or African American (n = 114), Hispanic or Latino (n = 869), Middle Eastern or North African (n = 204), White or Caucasian (n = 5,378), and Other (n = 519). BMI was determined based on self-reported weight and height. Weekly alcohol consumption was provided as one of: 0 drinks per week, 1-4 drinks per week, 4-7 drinks per week, 8–14 drinks per week, and 15 or more drinks per week. To calculate correlations to lifestyle, health, and demographics, lifestyle and health survey responses were scaled to a value between 0 (least healthy) and 1 (healthiest). Binary demographic variables were arbitrarily assigned either 0 or 1 and treated as factors during modeling and classification. The metadata for all 8,045 samples is included as Supplementary Table S1.

In addition, we used another cohort of 225 buccal samples which was collected independently and included responses for alcohol consumption, BMI, chronological age, smoking status, and race/ethnicity (Shokhirev et al., 2024), for independent validation of the predictors in this work. The metadata for all 225 samples is included as Supplementary Table S5. For the validation dataset, some respondents indicated whether or not they had five or more drinks in a single day within the past year.

### Sample collection

Volunteers were mailed a buccal collection kit, which consisted of two VARE (Shenzhen City, Guangdong, China) flocked swabs (cat. no. VF106-80), two Mawi DNA Technologies (Pleasanton, California, United States of America) iSWAB-Discovery Human DNA collection devices (cat. no. ISF-T-DSC), customized instructions, and mailing pouches as described previously (Shokhirev et al., 2024). 8,045 samples were returned and passed quality control checks.

### EPIC array

Samples were preprocessed at Tempus Labs (Peachtree Corners, Georgia, United States of America) according to Illumina’s (San Diego, California, United States of America) protocols for MethylationEPIC array preprocessing and loaded onto MethylationEPIC arrays as described previously (Shokhirev et al., 2024).

### EPIC preprocessing

Computational processing of data was carried out using the R programming language version 4.3.1 (https://www.r-project.org/). The raw idat files were preprocessed using the minfi (v 1.46.0) preprocessing pipeline (Aryee et al., 2014), starting from ∼850,000 CpGs. In short, datasets were read in using the read. metharray.exp function, Noob normalization (Triche et al., 2013) was applied. Cell type prediction was carried out using the EpiDISH package (v 2.14.1) (Zheng et al., 2019), using the centEPiFibIC and centBloodSub references and RPC method with maxit = 100,000 to estimate cell proportions. Epithelial, neutrophil, and the sum of B cells, natural killer cells, CD4-T cells, CD8-T cells, monocytes, and eosinophils (collectively called otherImmune) were calculated. Importantly, cell type proportion is not included as part of model training because cell-type proportions are derived from the methylation, because it would introduce further bias and complicate training reproducibility, and because we have previously seen those inputs be deprioritized for these data when training a regularized model (Shokhirev et al., 2024). Beta values were used to calculate M values for all CpGs according to:

$$M_{i}=log_{2}\left(\frac{beta_{i}}{1-beta_{i}}\right),$$

where ${\;M}_{i}$ is the ith M value, and $beta_{i}$ is the ith beta value bound to be between 0.00001, and 0.99999 to avoid infinities. M-values were used for training and subsequent analyses and were selected over beta values to help improve sensitivity to small changes near the extreme values. From the ∼850,000 measured CpGs, we calculated the Pearson’s correlation coefficient for each CpG to each variable (smoking, alcohol, BMI, and chronological age) and the top 10,000 correlated CpGs were used as inputs for training classifiers and regressors. For ethnicity, the union of the top 2,500 CpGs correlated with ‘Asian or Pacific Islander’ responses was combined with the top 2,500 CpGs correlated with ‘Hispanic or Latino’ responses, which was combined with the top 2,500 CpGs correlated with ‘Middle Eastern or North African’ responses, which was combined with the top 2,500 CpGs correlated with ‘White or Caucasian’ responses, which was combined with the top 2,500 CpGs correlated with a response of ‘Black or African American’, resulting in a final set of 10,633 top correlated CpGs used for classifier training.

### Training smoking and ethnicity classifiers

The scikit-learn python module (https://scikit-learn.org/stable/) (V 1.6.1) was used to train binary and multi-output classifiers for smoking and ethnicity using the RandomForestClassifier model. In order to determine the optimal hyperparameters, we used a grid search approach and tested all combinations of the number of trees (20, 50, 100, 200, 500, 1,000, and 2000), and the maximum depth of the decision trees (2, 3, 4, 5, 6, and 7 levels). For each combination, we used a 5-fold cross validation approach to determine the optimal average accuracy across all folds. The optimal hyperparameters (500 trees, max depth 5 for smoking, and 2000 trees, max depth 5 for ethnicity) were then used to train each classifier using a 5-fold cross validation approach and the accuracy was evaluated on the held-out data sets representing 5 test sets combined into one set across all samples. The final classifier using the optimal hyperparameters was trained on all of the data.

Confusion matrices and associated statistics were calculated using the confusionMatrix function of the R caret package (https://cran.r-project.org/web/packages/caret/index.html) (V 6.0–94), and visualized with the *ggplot2* R package (https://cran.r-project.org/web/packages/ggplot2/index.html) (V 3.5.1) with color scales determined by the row-normalized percentages.

### Training feed forward neural network regressors

While the categorical variables smoking and race/ethnicity were trained using random forest classification, continuous variables such as weekly alcohol consumption, BMI, and chronological age were trained using feed forward neural networks (FFNN), a type of deep learning network that connects the inputs (top correlated CpGs) to the output through one or more hidden neural layers. We used the pytorch python framework (V 2.6.0) to design and train the FFNNs using an Adam optimizer with learning rate 1e-6 and 2,500 epochs unless otherwise stated and a 5-fold cross validation approach. The accuracy was evaluated on the held-out data sets representing 5 test sets combined into one set that included all samples. The learning rate was tuned manually to 1e-6 to allow for sufficiently slow optimization resulting in an asymptotic approach toward the minimum mean squared error by epoch 2,500. Final regressors were trained on the entire dataset.

For the alcohol and BMI regressors the FFNN included a linear layer mapping 10,000 CpGs to 2000 hidden neurons, followed by ReLU activation, followed by a second linear layer mapping 2000 hidden neurons to 500 hidden neurons, followed by another ReLU, followed by another linear layer mapping 500 hidden neurons to 50 hidden neurons, followed by a final ReLU filter, and a final linear layer mapping 50 hidden nodes to one output. The age regressors were constructed using different numbers of hidden layers.

Alcohol was predicted on a linear scale from 0 to 1 (0 = 15+ drinks per week, 1 = no drinks) but visualized as violin plots using the *vioplot* R package (https://cran.r-project.org/web/packages/vioplot/index.html) (V 0.4.0) and tested for significant differences between categories using Welch’s t-test.

For the explainable age regressors, the connections between the layers were masked according to known annotations of CpGs to genes in the EPIC annotation, and by the known annotations of genes to 1,532 Reactome (Milacic et al., 2024) pathways or 1,137 transcription factor target gene sets (Liberzon et al., 2015). The control FFNNs used fully connected layers with the same number of hidden nodes.

### Calculating importances of input features for classifiers and regressors

In order to dissect the potential underlying biology of each model, we calculated the importance of each CpG. For the classifiers, the importance of each input was calculated using the mean and standard deviation of accumulation of the impurity decrease within each tree using the feature_importances fitted attribute of the RandomForestClassifier class. For the regressors, Shap values were calculated using the DeepExplainer class of the python shap module (https://pypi.org/project/shap/) (V 0.47.2), using the first 100 samples to initialize the explainer. The shap values were calculated on the final regressor for all data and averaged across all samples to obtain average shap values for all input CpG features for each regressor. To calculate the top importances for CheekAge, which is a weighted average of 100 linear regression models trained on over 200,000 CpGs, we summed the weighted absolute values of all model weights for each model. Since model inputs were based on averaged inputs from clusters of CpGs, all CpGs in a cluster were given the same weighted weight. To compare the overlap between age regressors, we used the UpsetR R package (V 1.4.0) to visualize set intersections. All importances are included in Supplementary Table S7.

### Enrichment analysis for the most important classification and regression CpGs

In order to explore the potential underlying biology behind the most important classification and regression CpGs we subjected the top 1,000 of the most important CpGs to functional enrichment analyses using the missMethyl R package (V 1.34.0) (Phipson et al., 2016). We tested overrepresentation of genes annotated to the top 1,000 important CpGs for each classifier and regressor using both the transcription factor target gene database (1,115 gene sets) and the Reactome database v89 (1,736 gene sets) from the Molecular Signatures Database (Liberzon et al., 2015). The top 10 enriched terms are shown for each set of top CpGs with significant terms (FDR <0.1) highlighted. For the top 100 enrichment results for each model refer to Supplementary Table S3.

### Enrichment analysis for genes and genomic features

In addition to pathway and transcription factor target enrichment, we tested the top 1,000 important CpGs for gene enrichment, relation to CpG islands, and regulatory feature groups, as annotated by Illumina on the EPIC array manifest. Briefly, relation to CpG islands include the annotations: “Island”, “N_Shelf” (2–4 kb upstream), “N_Shore” (0–2 kb upstream), “OpenSea”, “S_Shelf” (2–4 kb downstream), and “S_Shore” (0–2 kb downstream). Regulatory feature groups include “Gene_associated”, “Gene_Associated_Cell_type_specific”, “NonGene_Associated”, “NonGene_Associated_Cell_type_specific”, “Promoter_associated”, “Promoter_associated_Cell_type_specific”, “Unclassified”, “Unclassified_cell_type_specific”, and unannotated. To test for significant enrichment of the feature, we counted the total number of each feature annotated to the top 1,000 most important age CpGs and calculated the probability of observing the count given the background distribution of counts for that feature across the entire array using the phyper cumulative hypergeometric distribution function in R. We report FDR corrected p-values as Supplementary Table S4.

### Testing for potential batch effects

Since samples were run in batches, it was important to check if significant batch effects were present in our dataset. To test for this, we performed principal component analysis (PCA) on each methylation dataset used for model training. Next, we used the methylation array plate ID to identify batches in our samples. To see if these batches cluster, we calculated the silhouette of each batch for the top 1,000 principal components using the *cluster* R package (https://cran.r-project.org/web/packages/cluster/index.html) (V 2.1.4) *silhouette* function on each of the methylation input datasets used for training each predictor. A positive silhouette value indicates that batches are clustering across one of the principal components with values close to 1 indicating perfect clustering. Therefore, batch effects would show up as positive clustering results across one or more principal components. Supplementary Table S2 lists all silhouette values for each dataset, demonstrating that none of the components for any of the input datasets show positive silhouette values.

### Calculating correlation of survey factors with delta age, smoking, BMI, and alcohol

To evaluate if delta age (predicted age - chronological age), predicted smoking, predicted BMI, and predicted alcohol consumption were significantly correlated with survey variables, we modeled each as a linear function of lifestyle/health factors, demographic factors, and technical factors using the *lm* R function:

delta age∼age+E+BMI+hf+af+st+sl+im+ed+so+di+ex+sex+sm+al+race,

pSmoking∼age+E+BMI+hf+af+st+sl+im+ed+so+di+ex+sex+sm+al+race,

pBMI∼age+E+BMI+hf+af+st+sl+im+ed+so+di+ex+sex+sm+al+race,

pAlcohol∼age+E+BMI+hf+af+st+sl+im+ed+so+di+ex+sex+sm+al+race,

where delta age is the deep learning predicted age minus the chronological age, pSmoking is the predicted smoking, pBMI is the predicted BMI, pAlcohol is the predicted alcohol, age is the chronological age, E is the epithelial cell proportion, BMI is the calculated body mass index, hf is self-rated health, af is self-perceived aging, st is stress level, sl is sleep quality, im is relative immune health, ed is education level, so is social satisfaction, di is the fraction of a diet that’s plant-based, ex is weekly exercise, sex is the predicted sex, sm is smoking status, al is alcohol habits, and race is self-reported race/ethnicity. The linear model statistics are included in Supplementary Table S6.

## Data Availability

The data analyzed in this study is subject to the following licenses/restrictions: The buccal methylomic dataset analyzed here is not publicly available. It can, however, be interrogated via the Shiny app CheekAge Explorer (https://cheekage.tallyhealth.com/). Requests to access this dataset should be directed to max@tallyhealth.com.
