# Supplementary material for: Using buccal methylomic data to create explainable aging clocks as well as classifiers and regressors for lifestyle and demographic factors
Source: Front Genet. 2025 Oct 1;16:1637186. doi: 10.3389/fgene.2025.1637186 (PMC12521809; doi:10.3389/fgene.2025.1637186)
Supplement: Supplementary file 4 [file Supplementaryfile1.docx]

Supplementary Figure and Table Descriptions

**Supplemental Figure S1**: **Validating trained predictors in another buccal dataset**. A) Scatterplot showing predicted age as a function of chronological age. Red line is the linear trend line, Gray line is the y=x line. B) Violin plots showing distribution of predicted alcohol abstinence (1 = no alcohol, 0 = 15+ drinks) for 6 categories of answers about alcohol consumption. Bars with p-values indicate significant differences using Welch’s T-test. Violin plot colors are different to help differentiate the different categories only. C) Scatterplot showing predicted BMI vs true BMI. Red line is the linear trend line, Gray line is the y=x line. D) Confusion matrix showing predicted smoking vs true smoking state. Table shows basic summary statistics. Percent values indicate row-normalized percentages and correspond to color bar on right. E) Confusion matrix of predicted race/ethnicity vs true race/ethnicity. Table below shows summary statistics overall and for each race/ethnicity category. Percent values indicate row-normalized percentages and correspond to color bar on right.

**Supplemental Figure S2: Linear additive models for predictions.** A-C) Barplots showing significance of linear association for predicted smoking (A), predicted BMI (B), and predicted alcohol consumption (C). D-F) Linear best fit coefficients for predicted smoking (D), predicted BMI (E), and predicted alcohol consumption (F). Colored bars represent significant linear associations. The variables: age is the chronological age, Epi is the epithelial cell proportion, BMI is the calculated body mass index, health is self-rated health, healthfeel is self-perceived aging, stress is stress level, sleep is sleep quality, sick is relative immune health, education is education level, social is social satisfaction, diet is the fraction of a diet that is plant-based, activity is weekly exercise, sex is the predicted sex with male set to 1 and female to -1 arbitrarily, smoke is smoking status, alcohol is alcohol consumption.

**Supplemental Figure S3: Number of hidden layers determines accuracy of age prediction results.** A-D) Models with specified layers were trained using a five-fold cross validation approach and held-out test values are plotted and evaluated for accuracy compared to chronological age. A) Simplified model with ReLu activation of chronological age directly. B) Model with one 2000 node hidden layer. C) Model with one 2000 node hidden layer and another 500 node hidden layer. D) Model with one 2000 node hidden layer, a 500 node hidden layer, and a 50 node hidden layer. RMSE: Root mean squared error, MAE: mean absolute error, R: Pearson’s correlation. Significance of the correlation is indicated by the p-value. Trend is the trendline equation.

**Supplemental Figure S4: Overlap analysis of the top 1,000 most important CpGs across models.** A) UpsetPlot showing overlap counts of the top 1,000 most important CpGs between the four age predictors described in Supplementary Figure S3. B) UpsetPlot showing overlap counts of the top 1,000 most important CpGs between all age models trained in this work.

**Supplemental Figure S5: A proof-of-concept, explainable deep learning model using transcription factor target gene sets.** A) Chronological age-correlated CpGs are connected to a hidden layer of genes annotated to those CpGs. Genes are connected to the Transcription factor target (TFT) gene sets they are a part of. TFT gene set neurons are then used to estimate chronological age. The model was trained using a five-fold cross validation approach and held-out test values are plotted and evaluated for accuracy compared to chronological age. B) A fully connected, unexplainable version of the model in A) was trained for comparison using a five-fold cross validation approach and held-out test values are plotted and evaluated for accuracy compared to chronological age. RMSE: Root mean squared error, MAE: mean absolute error, R: Pearson’s correlation. C) Top weighted pathways in the final explainable model. D) Example showing calculated weights for top TFT sets for a sample with a high delta age of +8.4 years and a chronological age of 45.0 years. E) Example showing calculated weights for top TFT sets for a sample with a low delta age of -8.8 years and a chronological age 91.2 years.

**Supplemental Table S1: Metadata for 8045 buccal samples.** This table includes all metadata for the 8,045 buccal methylation EPIC samples used for regressor and classifier training. It also includes all predicted values.

**Supplemental Table S2: Silhouette analysis for batch effects.** This table contains the mean silhouette values of batch ids across the top 1000 principal components of each of the methylation inputs tables. Values above 0 indicate clustering of batches, while values less than 0 indicate no clustering of batches for a particular component.

**Supplemental Table S3: Pathway enrichment analyses for 1,000 most important CpGs of each predictor.** This table shows the top 100 Reactome pathways and transcription factor target gene sets for each set of top 1,000 CpGs.

**Supplemental Table S4: Other enrichment analyses for 1,000 most important age CpGs.** This table shows the enrichment FDRs for the annotated genes, relation to island features, and genomic regulatory groups for the top 1,000 age CpGs. Significant FDRs indicate enrichment of the feature over genomics background using a hypergeometric test.

**Supplemental Table S5: Metadata for 225 independent buccal samples.** This table includes all metadata for the independent 225 buccal methylation EPIC samples used to validate the regressors and classifiers trained in this work.

**Supplemental Table S6: Linear model statistics for delta age, predicted smoking, predicted alcohol, and predicted BMI.** This file tabulates the linear model coefficients, statistics, and FDRs for four separate linear models assuming an additive relationship between the shown metadata variables and the predicted variable.

**Supplemental Table S7: Top 1,000 most important CpGs for all predictors and CheekAge.** This table shows the top 1,000 most important CpGs for each of the predictors. We also include the top 1,000 most important CpGs from our previously published CheekAge model.

**Data in Mendeley (DOI: 10.17632/m4zjkxss8f.2):** This contains all trained classifiers (.pkl extension) and regressors (.pth extension) as well as R and Python code used for pre/post processing and model training, respectively. It also includes the ordered feature names for the input CpGs to enable prediction on your own CpG dataset.
