# Supplementary figures and images for: Using buccal methylomic data to create explainable aging clocks as well as classifiers and regressors for lifestyle and demographic factors
Source: Front Genet. 2025 Oct 1;16:1637186. doi: 10.3389/fgene.2025.1637186 (PMC12521809; doi:10.3389/fgene.2025.1637186)

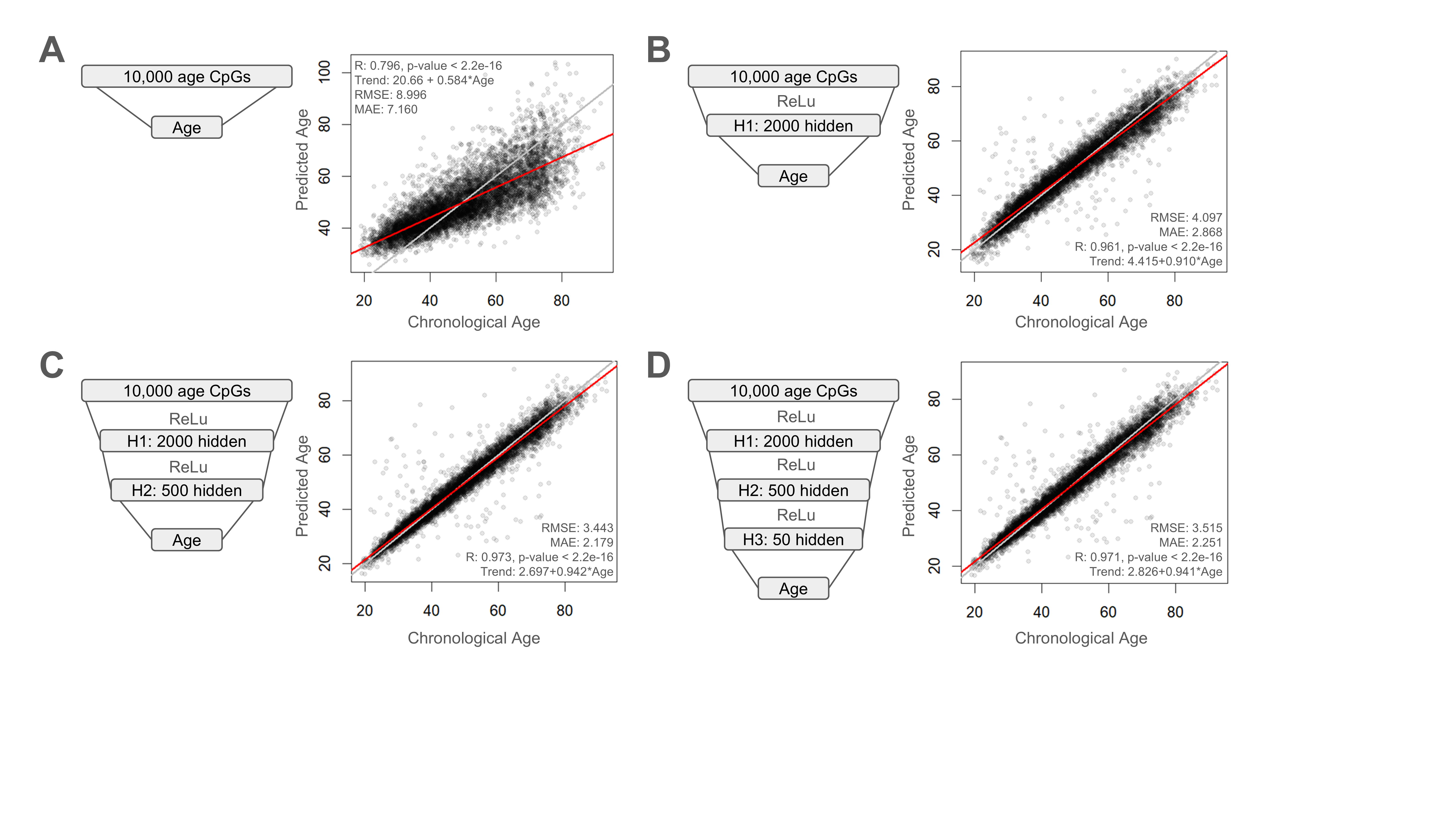

Supplement: Supplementary file 5 [file Image3.tif]

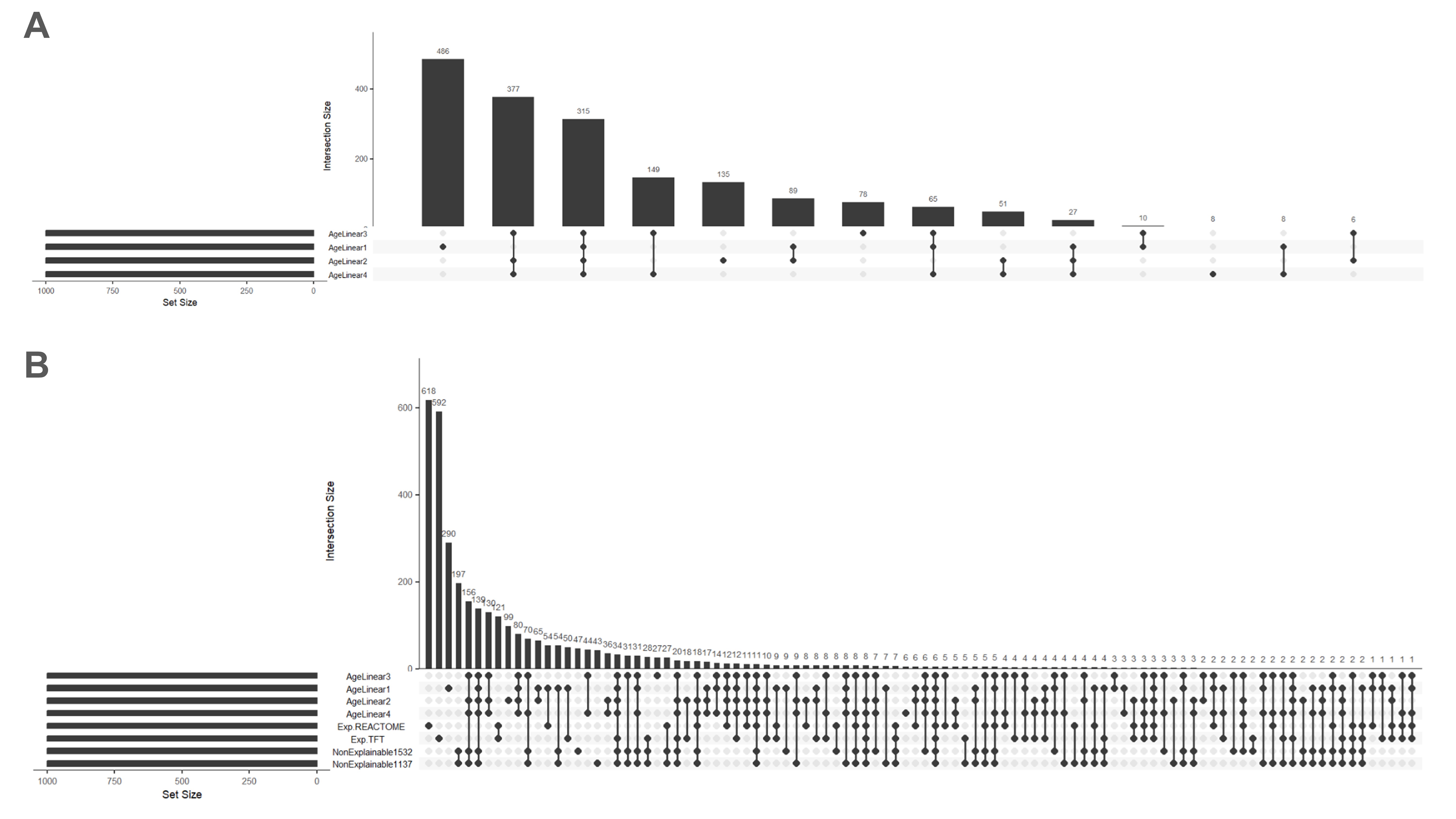

Supplement: Supplementary file 6 [file Image4.tif]

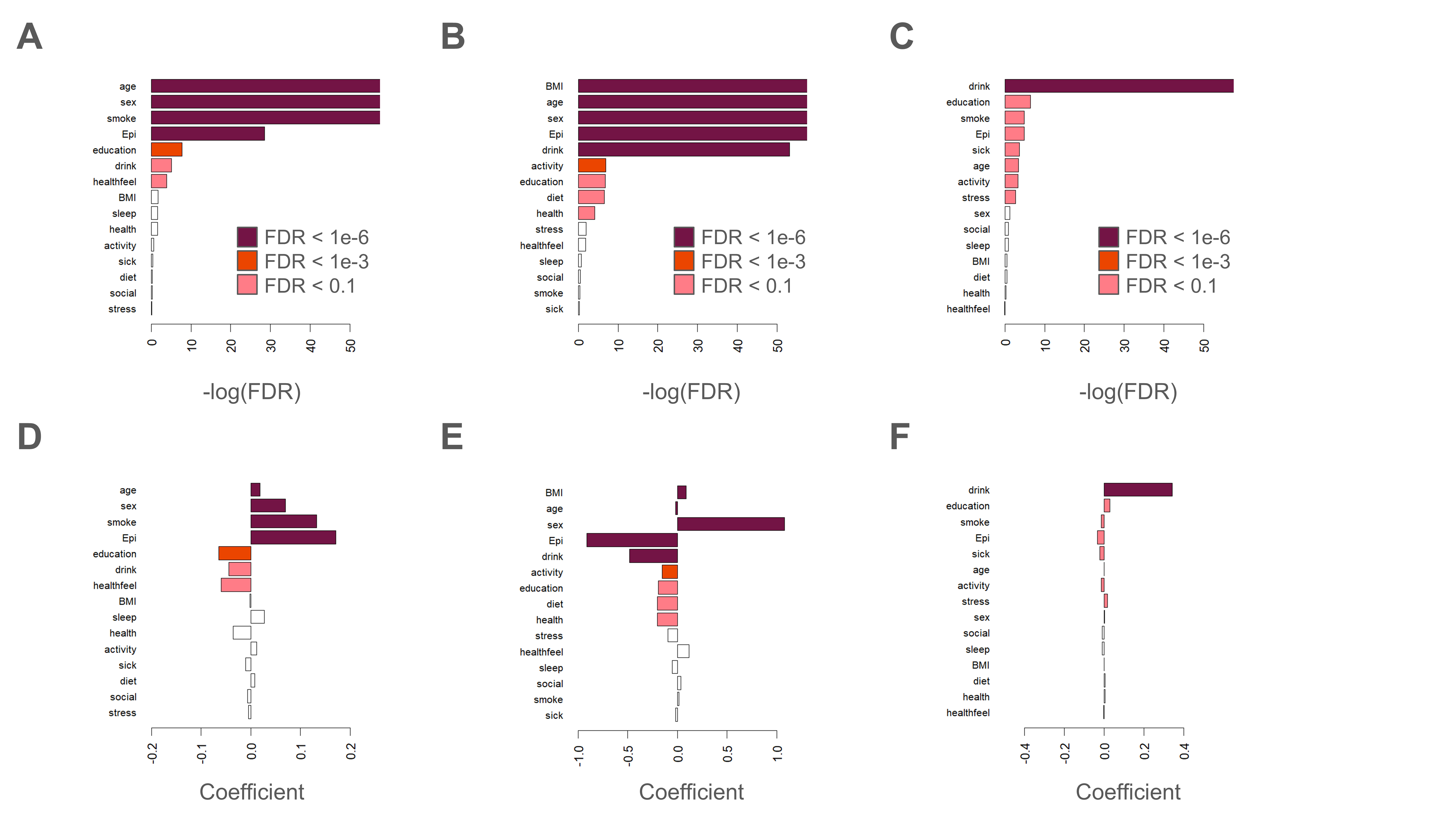

Supplement: Supplementary file 7 [file Image2.tif]

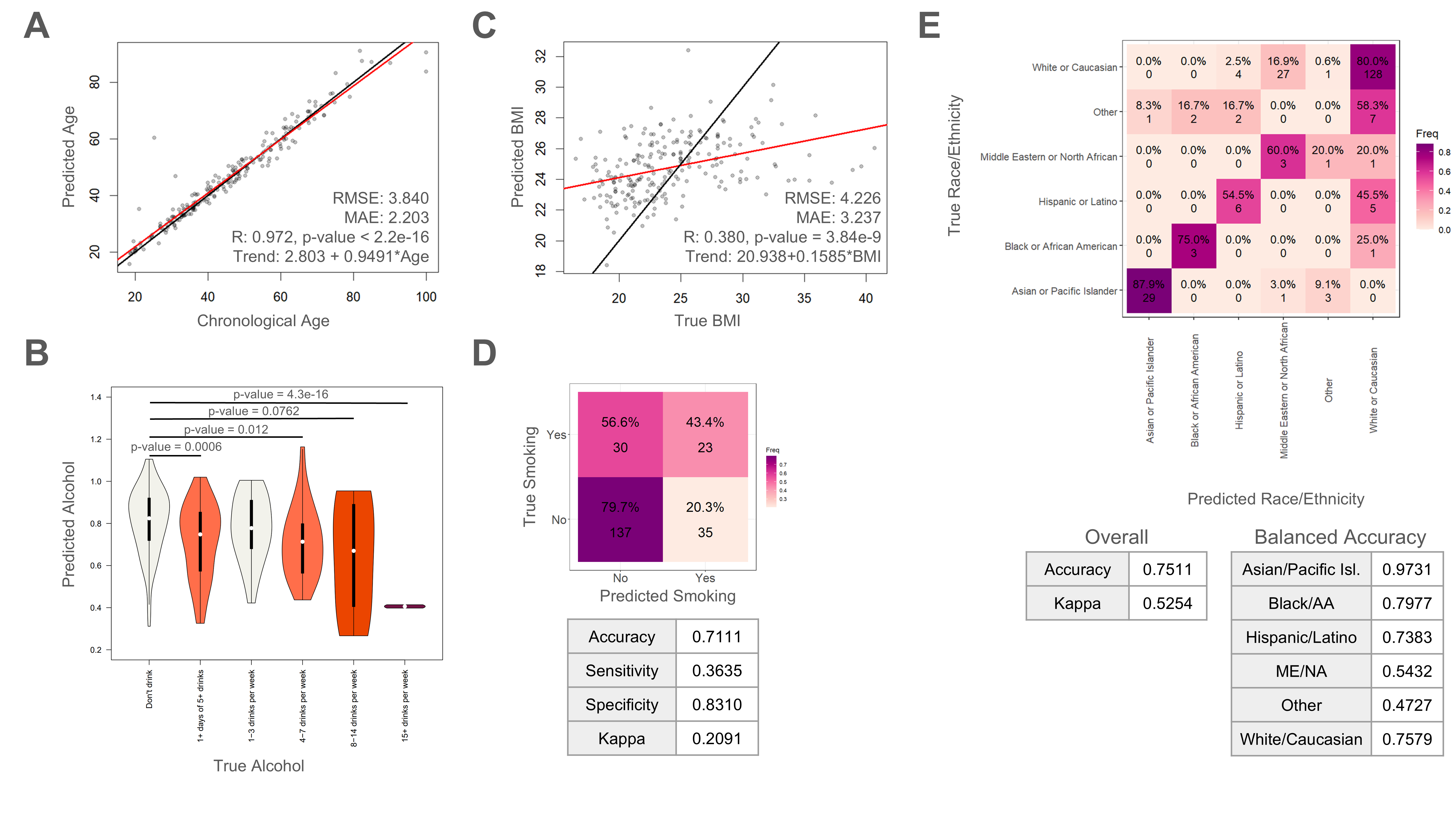

Supplement: Supplementary file 8 [file Image1.tif]

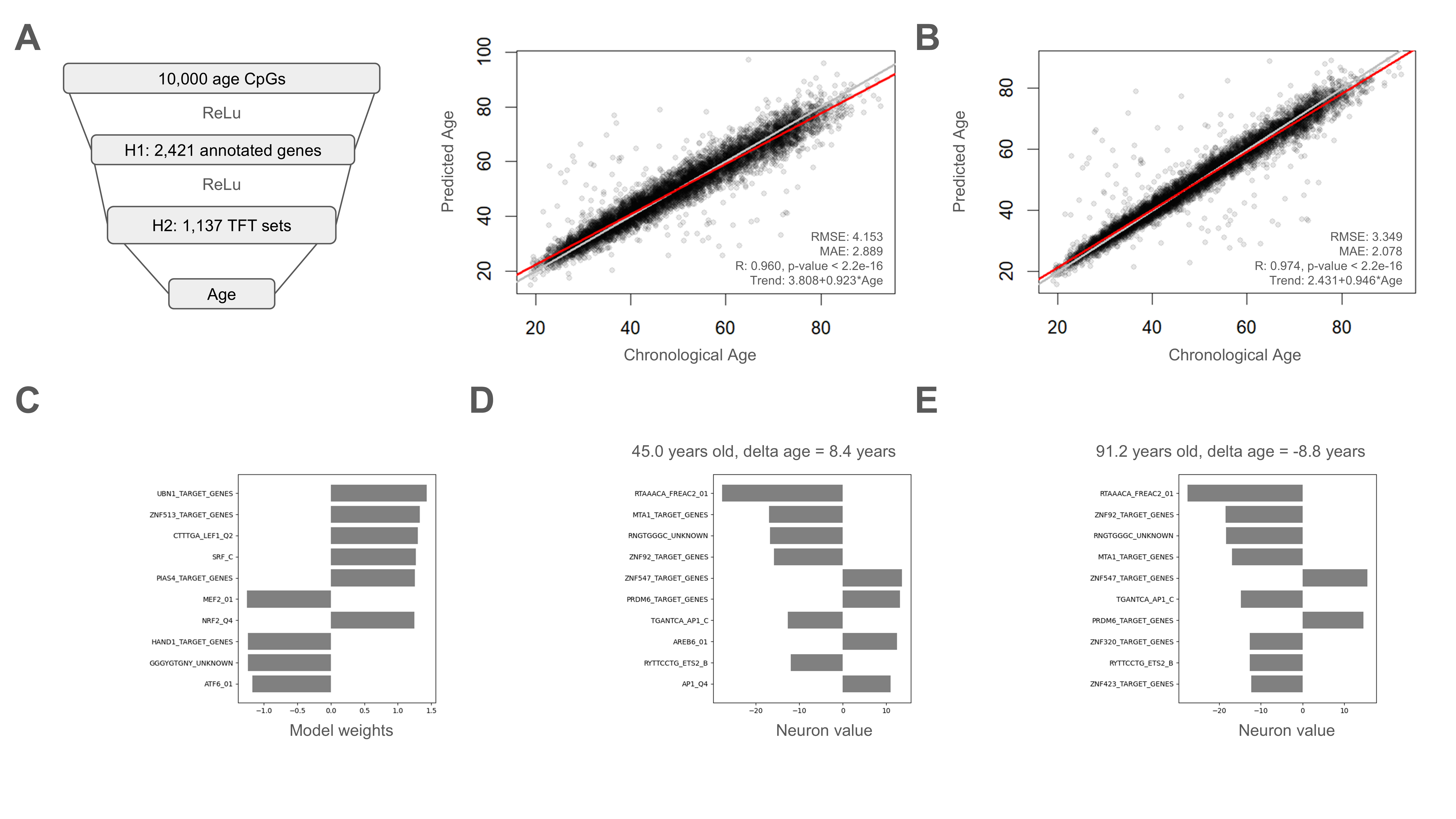

Supplement: Supplementary file 11 [file Image5.tif]
